# Th1 and Th2 Immune Response in Chronic Hepatitis B Patients during a Long-Term Treatment with Adefovir Dipivoxil

**DOI:** 10.1155/2010/143026

**Published:** 2010-11-29

**Authors:** Yanfang Jiang, Zhenhua Ma, Guijie Xin, Hongqing Yan, Wanyu Li, Huining Xu, Chunhai Hao, Junqi Niu, Pingwei Zhao

**Affiliations:** ^1^Department of Hepatology, First Hospital, Jilin University, Changchun 130021, China; ^2^Department of Pathology and Laboratory Medicine, Emory University, Atlanta, GA 30322, USA

## Abstract

Adefovir dipivoxil treatment has significantly improved the outcome of chronic hepatitis B virus (HBV) infection. However, it remains largely unknown how immune system responds to the treatment. Chronic HBV patients were treated with adefovir dipivoxil and examined for serum HBV DNA loads, cytokines, and T helper (Th1) and 2 (Th2) cytokine producing T cells during 104 weeks of the treatment. Th1/Th2 cytokines producing T cells were significantly lower in chronic HBV patients as compared to normal individuals. Adefovir dipivoxil treatment led to the increase of Th1/Th2 cytokines producing T cells and serum cytokine levels in association with the decline of HVB DNA load. In contrast, Th1/Th2 cytokines producing T cells remained lower in one patient detected with adefovir dipivoxil resistant HBV A181T/V mutation. This study has established inverse correlation of the increase of Th1/Th2 immunity and the decline of HBV DNA load in chronic HBV patients during adefovir dipivoxil treatment.

## 1. Introduction

Chronic HBV infection is one of the most common infectious diseases and contributes to a million death per year worldwide [1]. China is among the highly endemic countries with approximately 8% of the population being chronically infected with the virus [2]. In the last decade, clinical management of chronic HBV infection has significantly improved mainly due to the introduction of nucleoside and nucleotide analogs [3]. Lamivudine is the first approved nucleoside analog that has been clinically proved to be capable of inhibiting HBV replication, enhancing the seroconversion of hepatitis B e antigen (HBeAg) to antibody (HBeAb), and delaying the progression of the HBV-related complications [4–6]. However, prolonged treatment with lamivudine is limited by the emergence of HBV mutations and thereby drug resistance in up to 67% of patients [7]. 

Adefovir dipivoxil is a nucleotide analog of adenosine monophosphate [8, 9] that is converted intracellularly to the active metabolite, adefovir diphosphate, which can inhibit DNA polymerase of both wild-type HBV and lamivudine-resistant HBV mutant [10]. Randomized trials have proved the effectiveness of adefovir dipivoxil in treating HBeAg-positive [11–13] and HBeAg-negative chronic HBV patients [14, 15]. Adefovir dipivoxil resistance due to the mutations in the DNA polymerase of HBV [16] was observed only in 18% HBeAg-negative [17] and 20% HBeAg-positive chronic HBV patients treated up to four years [12]. In contrast, however, a multicenter randomized trial in Chinese patients has shown no drug resistance during the four years treatment [18]. 

T-cell immunity plays a critical role in determining the outcome of HBV infection [19]; however, it remains to be established how immune system responds to adefovir dipivoxil and thereby contributes to sustained viral control and improved liver function. In acute HBV infection, CD8 cytotoxic T and CD4 helper T-cells-mediated immunities are activated and involved in the clearance of HBV from the hepatocytes [20]. In chronic HBV infection, however, CD8 and CD4 T-cell immunity are hyporesponsive in association with persistent HBV serum load [21], which suggests that high HBV load may impair T-cell immunity and antiviral treatments can improve the immunity by reducing viral load [19]. In support of this, lamivudine therapy has been proven to reduce HBV serum load and restore CD4 T-cell immunity in chronic HBV patients [22, 23]. 

Recent studies have further shown that adefovir dipivoxil treatment leads to the seroclearance of HBV DNA and the recovery of CD4 [24, 25] but not CD8 T-cell immunity in chronic HBV patients [25]. Th1 cells are characterized by their capability of producing Th1 cytokines, interferon-*γ* (IFN-*γ*), interleukin-2 (IL-2), and tumor necrosis factor-*α* (TNF-*α*) whereas Th2 cells are able to synthesize the Th2 cytokines IL-4 and IL-10 [26]. In this paper, we report that a long-term treatment with adefovir dipivoxil leads to the decline of HBV DNA load and the increase of Th1/Th2 immunity in chronic HBV patients in China.

## 2. Patients and Methods

### 2.1. Patients

A total of 22 CHB (17 men and 5 women) patients who were presented to the Jilin University First Hospital and 20 healthy controls were included in the study. These patients were treated with adefovir dipivoxil (Gilead Science, Forster City, CA, USA) 10 mg orally once daily for 104 weeks. Th1 and Th2 cytokines including IL-2, IFN-*γ*, TNF-*α*, IL-4, and IL-10 were measured before and at 12, 24, 36, 52, 65, 78, 92, and 104 weeks after treatment. Viral suppression was evaluated by measurement of HBV DNA along with biochemical markers, AST and ALT. During the followup, one and then three patients dropped out, respectively, at the 36th and 78th weeks of the treatment. The study was approved by the First Hospital Ethical Committee of Jilin University and carried out according to the 1975 Declaration of Helsinki. All patients provided written informed consent prior to study enrollment.

### 2.2. Flow Cytometric Analysis of Intracellular Cytokine Staining (ICS)

Venous blood samples were collected from chronic HBV patients before (0 week) and after the treatment with adefovir dipivoxil for 12, 24, 36, 52, 65, 78, 92, and 104 weeks. Blood cells were stimulated and cytokine-secreting cells were analyzed using flow cytometry according to previously reported protocol [27]. For the analysis of intracellular cytokines production, 1000 *μ*L blood was diluted with Iscove's modified Dulbecco's medium (1:1 volume). The diluted whole blood was stimulated with phorbol 12-myristate 13-acetate (PMA; Sigma Chemical Co., St. Louis, MO) (50 ng/mL) plus 2 *μ*g/mL of ionomycin for 6 hours. 10 *μ*g/mL brefeldin A was added at 2 hours before the cells were collected and stained with antibodies. All antibodies were purchased from BD Biosciences (BD Bioscience, Heidelberg, Germany). After being stained with 10 *μ*L antibodies to surface markers (anti-CD3-PerCP, anti-CD8-FITC, or anti-CD8-PE). The cells were permeabilized and fixed using fixing reagent (Caltag, US) and rupture of membrane and the dissolution of blood reagent (Caltag, US) according to the manufacturer's instructions. The anti-IL-2-FITC, anti-TNF-*α*-FITC, anti-IL-4-PE, anti-IFN-*γ*-PE or isotope-matched control antibodies were added for 30 minute. All the samples were analyzed using a FACSCalibur instrument (FACSCalibur, Beckton Dickinson) and FlowJo software. At least 20,000 events per run were acquired.

### 2.3. Cytometric Bead Array of Serum Th1 and Th2 Cytokines

Serum cytokine levels were determined by cytometric bead array (CBA) [28], based on the manufacturer's protocol (CBA, BD Biosciences, San Joes, CA). The protocol was modified based on the earlier report to measure cytokines in 25 *μ*L  serum [29]. The amount of cytokines was quantified using the cytometric bead array kit on a FACSCalibur cytometry (BD Biosciences) equipped with CellQuestPro and CBA software (Becton Dickinson). 

### 2.4. Hepatitis Serology and HBV Mutation Analysis

Serum HBsAg, HBsAb, HBeAg, HBeAb, and hepatitis B core antigen antibody (HBcAb) were examined by commercial MURES Mikrotiterplatten enzyme immunoassays according to the manufacturer protocol (Abbott Laboratories, Abbott Park, US). HBsAg quantitation was performed by automated chemiluminescent microparticle immunoassay [30], based on the manufacturer's protocol (Abbott Laboratories, Abbott Park, IL). Serum HBV DNA was measured by quantitative PCR assay using luciferase quantitation detection kit (Roche Amplicor, limit of quantification 300 copies/mL). Serum HBV DNA was examined for mutations using HBV Drug Resistant Mutations Assay (Gene Tech Company Limited, Shanghai, China) [31]. In brief, serum HBV DNA was amplified by PCR and pyrosequenced to detect the following mutations of HBV polymerase: I169T, V173L, L180M, A181V/T, T184G/S/A/C, A194T, S202G/I, M204V/I, N236T, and M250V.

### 2.5. Statistical Analysis

All clinical and flow cytometry data were compared using Wilcoxon rank sum test and Chi-square test. SAS version 8.0 software was used. Correlations were determined using Spearman's correlation test. Results are given in median (range), unless specified otherwise. For all tests, value of *P* < .05 was considered statistically significant.

## 3. Results

### 3.1. Demographic Background

Twenty-two Chinese patients (17 men and 5 women) were enrolled in the study after initiating screening of HBV serology and liver biochemistry (Table 1). The ages of these patients ranged from 30 to 61 years old (average age at 45.9 ± 8.1) with 10.3 ± 1.6 years of clinical history of chronic HBV infection. Initial screening of serum hepatitis virology detected HBsAg, HbeAb, and HBV DNA in all patients with an average HBV DNA load at 5.8 ± 0.2log _10_  copies/ml. Biochemical analysis of serum revealed elevated ALT and AST, respectively, at the median of 27.6 (10.1–445.6) and 26.8 (7.1–312.4) units/L in all the patients. In contrast, twenty health volunteers were serologically negative for HbsAg, HbeAb, and HBV DNA and biochemically normal for ALT and AST.

### 3.2. Th1 and Th2 Cytokine Producing CD3+CD4+ T-Cells at Baseline in Chronic HBV Patients

The results revealed the baseline levels of Th1 and Th2 cells producing IL-2, IFN-*γ*, TNF-*α*, IL-4, and IL-10, respectively, at the median of 12.16, 5.73, 15.75, 5.69, and 11.7 in the health volunteers (Table 2). In contrast, however, both Th1 and Th2 cytokine-producing CD3+CD4+ T-cells were detected at very lower levels in the chronic HBV patients with the median of cytokine-producing cells at 0.6 (IL-2), 0.4 (IFN-*γ*), 0.7 (TNF-*α*), 0.4 (IL-4), and 0.6 (IL-10). This study further confirms that both Th1 and Th2 immunity are functionally impaired in chronic HBV patients.

### 3.3. Response of Th1/Th2 Cytokine-Producing T-Cells to Adefovir Dipivoxil Treatment

**Figure 1:**
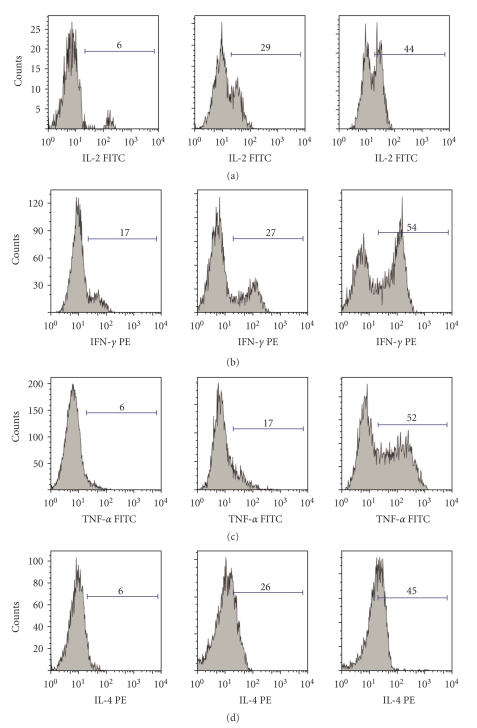
Analysis of intracellular cytokine staining of IL-2, IFN-*γ*, TNF-*α*, and IL-4 from gated CD3+CD4+, respectively.

CD3+CD4+ T-cells were gated and examined by intracellular staining of Th1 (IL-2, IFN-*γ*, TNF-*α*) and Th2 cytokines (IL-4, IL-10) (Figure 1). Prior to adefovir dipivoxil treatment, Th1 (IL-2, IFN-*γ*, TNF-*α*) and Th2 cytokines (IL-4, IL-10) producing CD3+CD4+ T-cells were significantly lower in chronic HBV patients as compared to healthy individuals (Table 2). Four patients dropped out the study. Of the remaining eighteen patients in the study, sixteen showed no HBV mutations, whereas one had lamivudine resistant HBV M204V/I mutation and another showed adefovir dipivoxil resistant HBV A181T/V mutation. Adefovir dipivoxil treatment resulted in the increase of both Th1 and Th2 cytokines-producing CD3+CD4+ T-cells in all the patients without HBV mutations throughout the treatment (Table 3). Statistic analysis with SAS 8.0 software revealed a significant relationship (*P* < .05) between the increase of Th1/Th2 cytokine-producing cells and the decrease of HBV DNA loads, ALT, AST during the treatment (Table 4).

The levels of Th1 cytokines (IL-2, TNF-*α*) producing cells in the patients without HBV mutations reached the peaks at the 36th week of the treatment and started to drop and maintained the levels of normal healthy individuals at the 65th week of the treatment (Table 3). The peak of the number of Th2 cytokine, IL-4 producing cells was at the 65th week and started dropping to the normal individual levels approximately at the 78th week of the treatment (Table 3). The patient with the lamivudine resistant HBV M204V/I mutations showed a similar Th1/Th2 cytokine response with the nonmutation patients; however, the patient with adefovir dipivoxil resistant HBV A181T/V mutations presented with persistent lower levels of Th1/Th2 cytokines producing CD3+CD4+ T-cells.

### 3.4. Serum Th1 and Th2 Cytokine Response during Adefovir Dipivoxil Treatment

Our results have showed that the levels of intracellular cytokines were significantly lower in chronic HBV patients as compared to healthy individuals at baseline (Table 3), and increased during adefovir dipivoxil treatment (Table 4). In order to know the Th1/Th2 cytokines better, we further examined the same cytokines in the serum using cytometric bead array (CBA) and analyzed the data by Chi-Square Test. The results showed that there was a significant increase in the serum levels of Th1 (IL-2, IFN-*γ*, TNF-*α*) and Th2 cytokines (IL-4, IL-10) in the patients after the adefovir dipivoxil treatment as compared to the cytokine levels at the baseline. IFN-*γ* showed much more increase in the response to the adefovir dipivoxil treatment as compared to other four cytokines. The levels of Th1/Th2 cytokines producing cells reached the peaks at the 78th week of the treatment and maintained up to the 104th week of the treatment (Table 6). The levels and changes of serum cytokines were not associated with HBV DNA loads, ALT, and AST (*P* > .05).

### 3.5. Biochemical and Virological Response to Adefovir Dipivoxil Treatment

**Figure 2:**
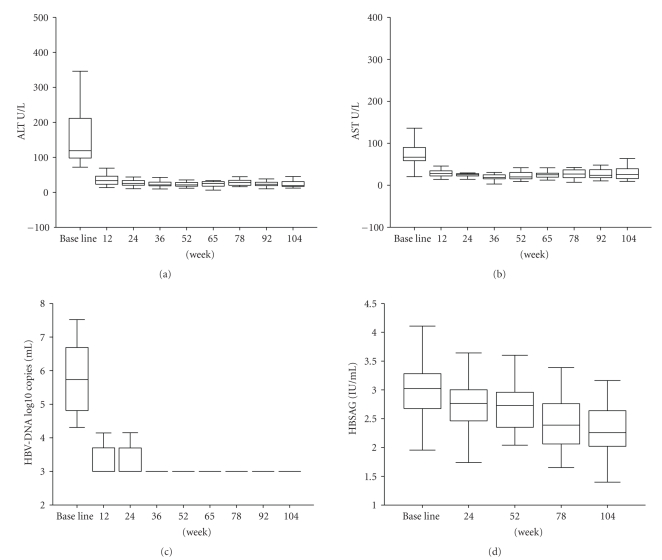
Biochemical and virological responses after adefovir dipivoxil treatment. ALT (a), AST (b), HBV-DNA loading (c), and HBsAg levels (d) were examined in the serum samples from chronic HBV patients at adefovir dipivoxil treatment week of 0 (baseline), 12, 24, 36, 52, 65, 78, 92, and 104 (*P* value for all treatment were statistically different from baseline <.05).

Biochemical analysis of the patients showed that ALT and AST started to decline soon after the treatment became normalized between 12th to 36th weeks of the treatment and remained normal (Figure 2). Elevated serum ALT and AST, however, remained in one of the patients through the 104 weeks of adefovir dipivoxil treatment. Serum HBsAg showed the tendency of decline. 

**Figure 3:**
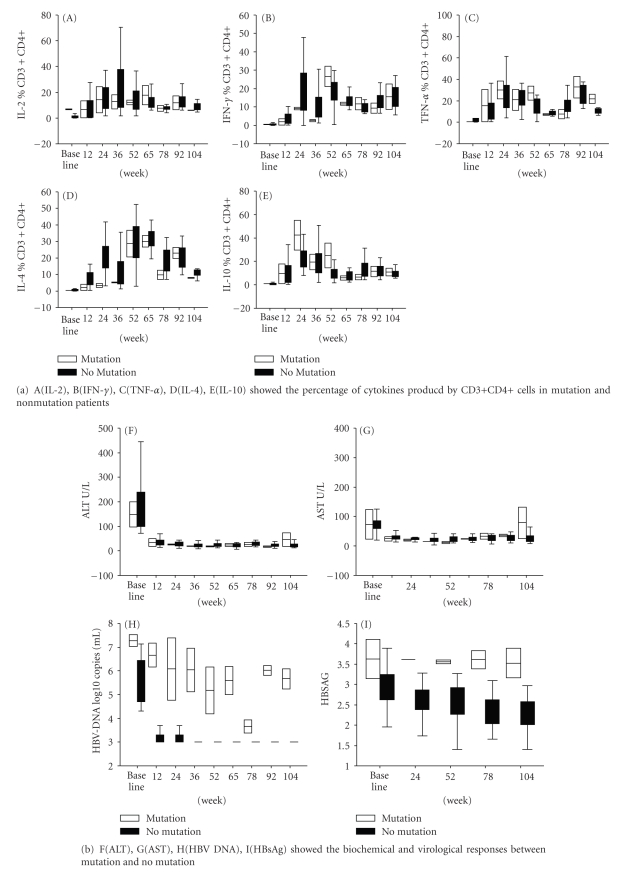
Analysis of Th1/Th2 cytokine producing CD3+CD4+ cells at adefovir dipivoxil treatment week (TW) between mutation and no mutation.

A basal serum HBV DNA load was high in all the patients, but decreased rapidly following the adefovir dipivoxil treatment. HBV DNA load dropped to the levels below the detection limit (<300 copies/ml), respectively, at 12 and 24 week treatment in 20 of 22 patients (Figure 2). Two remaining patients had detectable levels of HBV DNA throughout 104 weeks treatment. The serum samples of these two patients were examined for ten HBV mutations (I169T, V173L, L180M, A181V/T, T184G/S/A/C, A194T, S202G/I, M204V/I, N236T, M250V) that have been reported in the reverse transcriptase regions of HBV polymerase gene in association with the HBV resistance to the treatment of nucleoside and nucleotide analogs [17]. Of the two patients with consistently elevated HBV DNA load (Figure 3), one patient had HBV A181T/V mutations that are associated with adefovir dipivoxil resistance [11] and another patient had HBV M204V/I mutations of lamivudine resistance. Persistently elevated serum ALT and AST were detected in the patient with adefovir dipivoxil resistant HBV A181T/V mutations (Figure 3). All remaining patients without HBV mutations showed clear correlation between the decline of HBV DNA load and the normalization of ALT and AST through the 104 weeks of adefovir dipivoxil treatment (Figure 2).

## 4. Discussion

The introduction of nucleoside and nucleotide analogs has dramatically improved clinical management of chronic HBV infection, a major health issue in China. Large clinical trials of adefovir dipivoxil, in HBeAg-positive chronic HBV patients in China have proven that adefovir dipivoxil can significantly improve HBV serology and liver biochemistry after 48 and 52 weeks of treatment [18, 32]. HBeAg purportedly acts via interference with Th1/Th2 cross-regulation, and prevention of severe liver injury during adult infections [33, 34]. However, in this study, we have examined adefovir dipivoxil treatment of HBeAb-positive chronic HBV patients in China and shown that after 104 weeks of the long term treatment, twenty of twenty-two patients (90%) showed the seroclearance of HBV DNA and normalization of ALT and AST. These results are consistent with the earlier reports [18, 32] and further indicate that adefovir dipivoxil is a safe and effective therapeutic agent in a long-term treatment of chronic HBV patients in China. 

In the investigation of HBV mutations in adefovir dipivoxil resistance, we have identified HBV A181T/V mutations in one and M204V/I mutations in another of two patients that showed HBV DNA persistence through 104 weeks of adefovir dipivoxil treatment. HBV A181V/T and N236T mutations known to adefovir dipivoxil resistance in adefovir dipivoxil resistant patients [16, 35–37]. HBV A181T and N236T mutations have been reported in adefovir dipivoxil resistant patients in Taiwan [38] and Singapore [11]. Although these mutations were not reported in an earlier study of patients in China [18], we have detected HBV A181T/I mutations in one of our patients in the Chinese population. In addition, we have shown the persistent elevations of HBV DNA load and ALT and AST in this patient during adefovir dipivoxil long-term treatment. The HBV L180M and M204V/I mutations are associated with lamivudine resistance [17] and the concept that these mutations can be responsible for adefovir dipivoxil resistance is intriguing and remains to be confirmed in a sizable group of adefovir dipivoxil resistant patients. A recent study based in Hong Kong has indeed suggested that M204V/I mutation is associated with the resistance of HBeAg-positive chronic HBV patients to lamivudine and adefovir dipivoxil combination treatment [39]. 

It is generally believed that Chronic HBV infection is caused by the persistence of HBV covalently closed circular DNA (cccDNA) in hepatocytes [40], in which HBV cccDNA accumulates in the nuclei, persists as a stable episome, and serves as a template for the HBV mRNA transcription [41]. T-cell immunity is required for the clearance of cccDNA from hepatocytes through two immune mechanisms as established in acute HBV infection models: CD8+ cytotoxic T-cells clear HBV from hepatocytes through cytolysis of infected hepatocytes and regeneration of new hepatocytes [42] and Th1 and Th2 CD4+ T-cells-mediated humoral and cellular immunity is able to neutralize HBV by antibodies and inhibit HBV replication through cytokines [43]. In contrast, however, both CD8+ and CD4+ T immunities are functionally impaired in chronic HBV infection [21]. Here, we report that Th1 and Th2 T-cells-mediated immunity, as determined by the percentage of Th1 and Th2 cytokine producing cells in peripheral blood cells, are much weaker from chronic HBV patients than in those from health volunteers. The impaired Th1 and Th2 immunity are associated with the persistence of HBV load and the elevation of ALT and AST of blood samples from chronic HBV patients. 

In the study, we have further demonstrated the inverse correlation of the increase of Th1/Th2 cytokine producing T-cells and the decline of HBV DNA load in blood samples from chronic HBV patients during the long-term treatment with adefovir dipivoxil. It is well known that both CD8+ and CD4+ T-cell immunity are hyporesponsive in association with persistent HBV load in serum [21], which leads to the suggestion that high HBV load may impair T-cell immunity and antiviral treatments can improve T-cell immunity by reducing viral load [19]. Recent studies have shown indeed that adefovir dipivoxil treatment leads to the seroclearance of HBV [44] and the recovery of CD4 T-cell immunity [24, 25]. These studies suggest the possibility that adefovir dipivoxil, once converted intracellularly to adefovir diphosphate, inhibits HBV DNA polymerase, thus reduces HBV DNA load and thereby contributes to the recovery of T-cell-mediated cellular immunity.

## 5. Conclusion

This study has clearly shown that the long-term treatment with adefovir dipivoxil leads to the seroclearance of HBV DNA, normalization of ALT and AST, response of Th1 and Th2 cell immunity, and increase of serum Th1 and Th2 cytokines in chronic HBV patients and therefore suggested that adefovir dipivoxil treatment contributes to the reduction of HBV DNA load and the recovery of T-cell-mediated immunity in chronic HBV patients.

## Figures and Tables

**Table 1:** Demographic profiles of study participants.

Parameters	Chronic HBV patients	Health volunteers
No.	22	20
Age (years)		
Mean ± SD	45.9 ± 8.1	38.5 ± 11.8*
Median	46	36.5
Range	30–61	25–58
Sex N(%)		
Male	17 (77.3)	14 (70)
Female	05 (22.7)	6 (30)
Years from HBV diagnosis		
Mean ± SD	10.3 ± 1.6	NA
HBV DNA (log10 copies/ml)		
Mean	5.8	NA
SD	0.2	
ALT (U/L)		
Median	27.6	15.4*
Range	10.1–445.6	5.6–37.5
AST (U/L)		
Median	26.8	11.3*
Range	7.1–312.4	2.5–25.4
HbsAg (log _10_ IU/ml)		
Median	1082.5	NA
Range	90.0–12795.0	

*Differences between treatment groups and health volunteers were statistically significant (*P* < .5).

**Table 2:** Comparison of intracellular Th1/Th2 cytokines in chronic HBV patients and healthy volunteers.

Parameters (% CD3+CD4+)	Patients (no. 22)	Healthy volunteers (no. 20)	*P* value
IL-4	0.4 (0–6.0)	5.69 (1.99–14.22)	<.0001
IFN-*γ*	0.4 (0–9.4)	5.73 (3.78–12.09)	<.0001
IL-2	0.6 (0–7.0)	12.16 (4.44–17.82)	<.0001
IL-10	0.6 (0–3.8)	11.70 (6.87–21.32)	<.0001
TNF-*α*	0.7 (0.1–14.3)	15.75 (7.25–27.41)	<.0001

The data were expressed as median % and range.

**P value for all treatment were statistically different between patients and healthy volunteers (<.0001).

**Table 3:** Th1/Th2 cytokines producing CD3+CD4+ cells in patients.

TW	IL-2% CD3+CD4+	IFN-*γ*% CD3+CD4+	TNF-*α*% CD3+CD4+	IL-4% CD3+CD4+	IL-10% CD3+CD4+
TW0	0.6 (0–7.0)	0.4 (0–9.4)	0.7 (0.1–14.3)	0.4 (0–6.0)	0.6 (0–3.8)
TW12	3.9 (0.2–27.6)	3.0 (0.1–48.5)	8.6 (0.5–42.0)	6.6 (0.4–16.3)	6.5 (0.3–38.7)
TW24	11.6 (1.6–37)	22.5 (0.0–47.9)	24.7 (4.1–76.1)	17.2 (2.2–41.7)	21.0 (7.9–58.1)
TW36	14.7 (1.7–70.6)	7.4 (1.3–31.4)	22.9 (2.6–73.9)	5.9 (1.2–35.7)	19.7 (2.1–74.5)
TW52	11.4 (1.7–36.5)	19.6 (0.4–53.3)	15.5 (0.6–47.0)	34.4 (2.9–52.3)	9.6 (1.7–35.7)
TW65	10.2 (6.2–27.9)	12.5 (8.5–28.5)	8.1 (5.7–21.6)	32.9 (9.4–42.9)	7.7 (2.4–14.4)
TW78	7.6 (4.2–17.2)	9.8 (6.4–15.0)	14.9 (3.2–39.9)	13.6 (7.1–32.4)	9.7 (4.2–38.2)
TW92	14.0 (6.0–26.7)	11.9 (6.6–23.0)	26.2 (12.7–42.6)	21.1 (9.8–33.3)	12.0 (4.2–29.4)
TW104	7.4 (4.6–35.3)	14.2 (5.7–27.1)	10.8 (6.2–36.2)	10.6 (6.2–19.6)	9.4 (5.6–17.2)

*The data were expressed as median % and range.

**P value for all treatment were statistically different from TW0 <.05.

**Table 4:** Correlation between intracellular Th1/Th2 cytokines and ALT, AST, HBV DNA load, and HBsAg.

	IL-2% CD3+CD4+	IFN-*γ*% CD3+CD4+	TNF-*α*% CD3+CD4+	IL-4% CD3+CD4+	IL-10% CD3+CD4+
ALT					
*R*	−0.365	−0.402	−0.35	−0.386	−0.426
*P*	<.0001	<.0001	<.0001	<.0001	<.0001
AST					
*R*	−0.376	−0.356	−0.351	−0.285	−0.461
*P*	<.0001	<.0001	<.0001	<.0001	<.0001
HBVDNA					
*R*	−0.319	−0.382	−0.216	−0.479	−0.249
*P*	<.0001	<.0001	0.0034	<.0001	0.007
HBsAg					
*R*	−0.245	−0.186	−0.173	−0.223	−0.152
*P*	0.014	0.062	0.083	0.025	0.128

**Table 5:** Comparison of serum cytokine in chronic HBV patients and healthy volunteers.

Cytokine (pg/mL)	Patients (no. 22)	Healthy volunteers (no. 20)	*P* value
IL-4	4.5 (2.2–6.5)	3.0 (1.4–14.9)	0.016**
IFN-*γ*	5.85 (1.6–8.8)	4.4 (1.8–84.5)	0.009**
IL-2	5.5 (3.5–7.3)	2.3 (0.6–10.1)	0.044**
IL-10	5.7 (3.4–10.5)	1.6 (1.0–28.4)	0.499
TNF-*α*	2.9 (1.5–3.5)	1.6 (0.6–21.8)	0.032**

*The data were expressed as median % and range.

**P value for all treatment were statistically different between patients and healthy volunteers (<.05).

**Table 6:** Serum cytokine levels in the patients following adefovir dipivoxil treatment.

	IL-2 (pg/ml)	IFN-*γ*(pg/ml)	TNF-*α*(pg/ml)	IL-4 (pg/ml)	IL-10 (pg/ml)
TW0	5.5 (3.5– 7.3)	5.85 (1.6–8.8 )	2.9 (1.5–3.5)	4.5 (2.2, 6.5)	5.7 (3.4–10.5)
TW24	6.9 (4.4–10.6)	7.6 (3.3–9.6)	3.4 (2.4–29.0)	7.6 (3.3–9.6)	7.4 (3.1–11.9)
TW52	8.4 (4.4–13.1)	13.1 (9.1–23.5)	3.3 (2.4–4.6)	7.1 (3.7–11.1)	7.4 (3.7–17.8)
TW78	9.05 (7.4–11.9)	15.8 (9.7–31.2)	4.6 (3.8–30.9)	7.9 (5.9–10.1)	7.55 (4.4–17.0)
TW104	8.6 (4.5– 11.0)	14.5 (10.0–22.5)	3.9 (3.0–6.1)	7.6 (5.3–19.6)	7.6 (5.3–19.6)

*The data were expressed as median % and range.

**P value for all treatment were statistically different from baseline <.05.
